# Development of a 3D-Printed Nanocarbon Electrode Modified with Bimetallic Nanoparticles for Enhanced Electrochemical Detection of Dopamine

**DOI:** 10.3390/mi17050545

**Published:** 2026-04-29

**Authors:** Claudia Cirillo, Mariagrazia Iuliano, Nicola Funicello, Salvatore De Pasquale, Maria Sarno

**Affiliations:** 1 Department of Physics “E.R. Caianiello”, University of Salerno, Via Giovanni Paolo II, 132-84084 Fisciano, Italy; 2 NANO_MATES Research Centre, University of Salerno, Via Giovanni Paolo II, 132-84084 Fisciano, Italy

**Keywords:** 3D-printed electrochemical sensor, AgPt bimetallic nanoparticles, dopamine biosensing, conductive PLA electrode, real human urine sample

## Abstract

The rapid and reliable detection of dopamine (DA) is crucial for clinical diagnostics and neurochemical research. Here, we present an advanced electrochemical sensor fabricated by integrating 3D printing technology with bimetallic nanomaterials to achieve high sensitivity, selectivity, and reproducibility. A conductive polylactic acid (PLA) electrode was 3D-printed and subsequently activated to expose electroactive carbon domains. The surface was then modified with AgPt bimetallic nanoparticles (NPs), synthesized via a one-step solvothermal method, and coated with Nafion^TM^ 117 to form the AgPt@A-3DPE sensor platform. Morphological and structural characterization confirmed the formation of uniform, quasi-spherical AgPt nanoparticles with excellent dispersion. The sensor exhibited outstanding electrochemical performance, including a wide linear detection range for DA (0.5–100 µM), a low limit of detection (LOD) of 0.037 µM, and a significantly enhanced electroactive surface area (1.04 cm^2^). Furthermore, it demonstrates high selectivity in complex matrices, with minimal interference from common biomolecules such as ascorbic acid, uric acid, and glucose. Moreover, the practical applicability of the AgPt@A-3DPE sensor was successfully validated through the analysis of real human urine samples. This work demonstrates a low-cost, scalable, and highly efficient sensing approach, opening new avenues for personalized diagnostics and real-time monitoring of neurotransmitters in biomedical applications.

## 1. Introduction

Dopamine (DA) is a key neurotransmitter that plays a vital role in the central nervous system, being involved in several physiological functions such as memory, learning, endocrine regulation, motor control, and cognition [1]. Abnormal concentrations of DA in biological fluids have been strongly correlated with various neurological and psychiatric disorders, including learning disabilities, mood disorders, Parkinson’s disease, and Huntington’s disease [2]. Therefore, the accurate detection of DA is critical for the early diagnosis of these conditions. Several techniques have been developed for DA quantification, such as mass spectrometry, chromatography, and fluorescence spectroscopy [3,4]. However, these conventional approaches often suffer from limitations such as high cost, time-consuming procedures, and complex sample preparation. Compared to traditional techniques, electrochemical methods offer several advantages, including operational simplicity, high sensitivity, and excellent reproducibility, making them increasingly attractive to the analytical chemistry community [5,6]. During the electrochemical detection of dopamine (DA), the application of an external potential induces the transfer of two electrons from the o-phenolic hydroxyl groups of DA to the electrode surface, resulting in its oxidation to dopamine quinone. This redox process generates a characteristic electrochemical signal that is detected by differential pulse voltammetry (DPV). Nevertheless, significant challenges remain, particularly the need to achieve sufficient sensitivity to reliably detect DA at its low physiological concentrations in biological fluids [7] and addressing signal interference from coexisting species such as ascorbic acid (AA) and uric acid (UA), which can hinder accurate DA quantification. Therefore, the development of highly selective and sensitive detection strategies for DA remains a prominent research focus. In this context, 3D printing, also known as additive manufacturing (AM), has emerged as a promising and cost-effective technology in electrochemical applications [8,9]. It enables the fabrication of complex geometries with high precision, facilitating the large-scale production of devices such as batteries, wearable electronics, and electrochemical sensors [10]. Compared to conventional fabrication techniques, 3D printing significantly reduces production complexity and costs, while offering enhanced material versatility and design freedom. Among the various 3D printing techniques, Fused Deposition Modeling (FDM) is particularly attractive for sensor fabrication due to the favorable mechanical and electrical properties of its printed materials. FDM-based 3D-printed electrodes (3DEs) are typically composed of conductive composites embedded within non-conductive thermoplastic matrices such as acrylonitrile butadiene styrene (ABS) or polylactic acid (PLA) [11]. To improve the conductivity of these electrodes, various surface pretreatment strategies have been employed, including activation and modification steps [12]. These treatments remove insulating polymers and expose active sites, thereby enhancing electrochemical performance [13,14,15]. Notably, the combination of chemical and electrochemical treatment has been shown to significantly boost the activity of 3DEs [16]. To further improve sensor performance, noble metal nanoparticles (NPs) such as gold (Au), platinum (Pt), palladium (Pd), and silver (Ag) are widely used as electrode modifiers [17,18]. Among them, Pt is especially favored due to its superior electrical conductivity and electrocatalytic efficiency [19,20]. However, the high cost and limited natural availability of Pt [21] have prompted efforts to reduce its usage by forming bimetallic NPs with secondary metals such as Ag, Cu, Fe, and Ni. These bimetallic systems exhibit enhanced electrochemical properties owing to synergistic interactions between the constituent metals [22]. In particular, silver (Ag) is considered an excellent partner for Pt due to its biocompatibility, abundance, low cost, electrochemical stability, and sustainable catalytic activity [23].

In this study, we present an innovative 3D-printed electrochemical sensor for the detection of dopamine (DA). The electrodes were fabricated using FDM technology and a commercial conductive carbon black/polylactic acid (CB/PLA) filament (ProPasta) as the base material. Bimetallic AgPt NPs, synthesized via a solvothermal method and functionalized with a citric acid coating, were subsequently deposited onto the surface of the 3D-printed electrodes (3DPE), resulting in the AgPt@A-3DPE sensor. The morphological, structural, and electrochemical properties of AgPt@A-3DPE were thoroughly investigated. The sensor demonstrated good performance in terms of selectivity, stability, and a low limit of detection (LOD) for dopamine, with performance comparable to or exceeding previously reported electrochemical DA sensors.

## 2. Experimental Section

### 2.1. Materials

Protopasta (Vancouver, WA, USA) offers a commercial conductive PLA filament for 3D printing. N,N-dimethylformamide (DMF), sulfuric acid (H_2_SO_4_), sodium chloride (NaCl), potassium chloride (KCl), potassium ferricyanide (K_3_[Fe(CN)_6_]), potassium ferrocyanide (K_4_[Fe(CN)_6_]⋅3H_2_O), anhydrous disodium hydrogen phosphate (Na_2_HPO_4_), acetone, anhydrous sodium dihydrogen phosphate (NaH_2_PO_4_), silver nitrate (AgNO_3_), Potassium (IV) hexachloroplatinate (K_2_PtCl_6_), Dopamine (DA) and Nafion^TM^ 117 containing solution were purchased from Sigma-Aldrich (St. Louis, MO, USA).

### 2.2. Synthesis of AgPt NPs

AgPt NPs were synthesized through a solvothermal approach, following a modified procedure based on previously reported methods [24]. In a typical synthesis, K_2_PtCl_6_ and AgNO_3_ were dissolved in 40 mL of ethylene glycol at a molar ratio of 1:2 (Pt:Ag). The mixture was subjected to ultrasonication until a clear and homogeneous solution was obtained, ensuring complete dissolution of the metal precursors. Subsequently, 1.8 g of urea was added as a reducing and stabilizing agent, along with 0.1 g of citric acid, which acted as a capping agent to control nanoparticle growth and prevent aggregation. The resulting mixture was vigorously stirred for 30 min to facilitate precursor interaction and initiate nucleation. The solution was then transferred into a Teflon-lined stainless-steel autoclave and subjected to thermal treatment at 200 °C for 4 h to promote the complete formation of bimetallic PtAg nanoparticles under high-pressure conditions. After the reaction was completed and the system cooled to room temperature, the obtained nanoparticles were collected by centrifugation (7500 rpm, 30 min), thoroughly washed several times with ethanol to remove residual reagents and byproducts, and finally dried at 60 °C for 24 h to obtain a fine powder.

### 2.3. Preparation of the AgPt NPs Ink

For the preparation of the AgPt-based inks, AgPt nanoparticles (AgPt NPs) were dispersed in a 6% (*w*/*v*) ethanolic solution. To achieve a homogeneous dispersion of the nanoparticles and prevent agglomeration, the resulting mixture was ultrasonicated for 5 min using an ultrasonic probe (Hielsher UP400 S; Hielscher Ultrasonics GmbH, Teltow, Germany) [25].

### 2.4. Electrode Printing and Activation

Conductive PLA electrodes were fabricated using fused deposition modeling (FDM) on a Raise3D printer (Raise3D, Irvine, CA, USA). The process utilized a commercially available Protopasta conductive PLA filament as the raw material. The electrode design was created using Ideamaker software. Each electrode had a total length of 1.6 cm and a thickness of 1.5 mm, consisting of a circular base with a diameter of 6 mm and a 1 cm-long tail extending from it. Specific temperature conditions were applied during the printing process: the print bed temperature was set to 60 °C, the nozzle temperature to 205 °C, and the 3D-printed electrode, referred to as 3DPE, was obtained.

**Figure 1:**
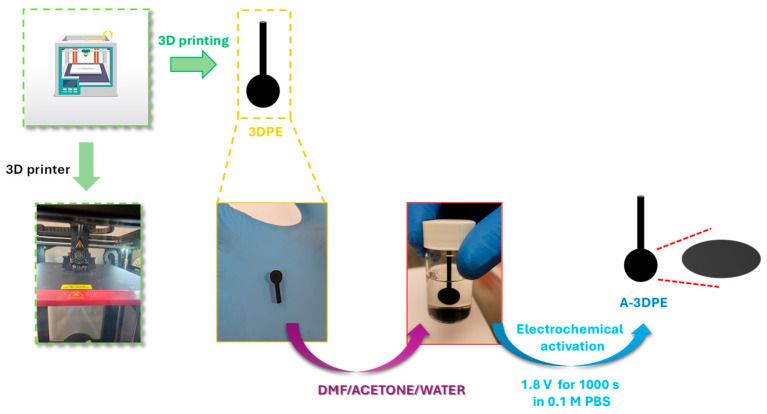
Schematic illustration of the preparation and activation process of the 3D-printed electrode (3DPE) to obtain the activated electrode (A-3DPE).

Post-processing treatment was employed to enhance the conductivity of the printed electrode [26,27]. The 3D-printed electrode was first immersed in 5 mL of DMF and sonicated for 1 min in an ultrasonic bath at full power. It was then rinsed, immersed in 5 mL of acetone, and sonicated for another minute. After a second rinse, the electrode underwent a final cleaning step by sonication in 5 mL of deionized water for 1 min [28]. The electrode was subsequently dried in a vacuum oven at 60 °C for 1 h. Finally, the electrode was electrochemical activation using an Autolab PGSTAT302N Potentiostat/Galvanostat (Metrohm Autolab B.V., Utrecht, The Netherlands). The activation was performed in a 0.1 M PBS solution, applying a constant potential of 1.8 V for 1000 s [29]. After activation, the electrodes were thoroughly rinsed with ultrapure water and dried at room temperature. The final activated 3D-printed electrode, referred to as A-3DPE, was obtained through these processes. The schematic process is illustrated in Figure 1.

### 2.5. Preparation of 3D Printed Electrodes Using AgPt Ink

For the construction of the electrochemical sensor, 10 μL of the AgPt ink solution was drop-cast onto the circular surface of the activated 3D electrode and allowed to dry overnight at room temperature to ensure proper immobilization. Subsequently, 10 μL of a 1% (*w*/*v*) ethanolic solution of Nafion^TM^ 117 was drop-cast onto the same surface and left to dry for 5 min, followed by gentle air-drying for an additional minute. This procedure enabled the stable and functional fabrication of the electrochemical sensor for DA detection.

### 2.6. Characterization

The morphology of the electrodes at different stages of the sensor preparation was analyzed using a TESCAN VEGA LMH scanning electron microscope (SEM) (TESCAN, Brno, Czech Republic) coupled with an energy-dispersive X-ray Spectroscopy (EDX) probe. Transmission electron microscopy (TEM) analysis was also performed using an FEI Tecnai electron microscope (FEI Company, Hillsboro, OR, USA) operating at 200 kV, with a LaB_6_ filament as the electron source. A drop of the diluted sample in ethanol was deposited onto a TEM grid (carbon-coated copper; Agar Scientific Ltd., Stansted, UK) and dried before insertion into the TEM column. The electrodes at different preparation stages were further characterized by X-ray diffraction (XRD) using a Bruker D2 diffractometer (Bruker, Billerica, MA, USA) with Cu Kα radiation (λ = 1.5406 Å).

### 2.7. Contact Angle

Contact angle (CA) analysis is a widely used method for evaluating surface wettability. In this study, measurements were carried out using a Theta Flex optical tensiometer (Biolin Scientific, Gothenburg, Sweden), supplied by Nordtest, which employs a static video-based approach for precise angle detection. Distilled water was used as the test liquid. A 2 µL droplet was carefully dispensed onto the sample surface using a microliter syringe, and its interaction with the surface was recorded with a high-resolution CCD camera. o ensure data reliability, at least ten measurements were performed at different positions on each sample, with image acquisition at 0.04 s intervals. The CA serves as a key indicator of surface wettability: values below 90° indicate a hydrophilic surface, where the liquid tends to spread, whereas values above 90° correspond to a hydrophobic surface, where the droplet remains more compact.

### 2.8. Electrochemical Method

The electrochemical measurements were performed using an Autolab PGSTAT302N potentiostat/galvanostat with AgPt@A-3DPE as the working electrode, Ag/AgCl reference electrode, and a platinum wire counter electrode. The conductivity of AgPt@A-3DPE was first investigated by cyclic voltammetry (CV) using 10 mM [Fe(CN)_6_]^4−/3−^ redox couple in a 0.1 M KCl solution at a scan rate of 50 mV·s^−1^. Electrochemical impedance spectroscopy (EIS) was then conducted over a frequency range from 0.01 to 50 kHz. Different DA concentrations, ranging from 0.5 to 100 μM, were analyzed using differential pulse voltammetry (DPV) in a 0.1 M phosphate-buffered saline (PBS) solution at pH 6.5. Each concentration was conducted in triplicate. DPV measurements were performed over a potential range of −0.1 to 0.4 V at a scan rate of 50 mV/s. All electrochemical experiments were conducted at room temperature.

### 2.9. Analysis of the Oxidase-Mimetic Activity of AgPt NPs

The oxidase-like catalytic activity of AgPt nanoparticles was determined by measuring the absorbance of oxTMB at 652 nm. Specifically, the reaction mixture was prepared by adding 60 μL of 3,3′,5,5′-tetramethylbenzidine (TMB; 10 mM, dissolved in ethanol) and AgPt nanoparticles (1.0 mg/mL) to 2.0 mL of 0.2 M acetate buffer adjusted to pH 3.5. The resulting solution was incubated at 30 °C for 10 min. After incubation, UV–vis absorption spectra were recorded, the absorbance intensity at 652 nm was measured, and the corresponding color changes were documented photographically.

### 2.10. Practical Application of the AgPt@A-3DPE Electrode in Human Urine

The AgPt@A-3DPE electrode was employed as the working electrode for the electrochemical determination of dopamine (DA) in real human urine samples. Urine samples collected from different individuals were centrifuged before analysis and subsequently subjected to quantitative determination. 0.1 M phosphate-buffered saline (PBS, pH 6.5) was mixed with human urine in a 50:50 (*v*/*v*) ratio and spiked with standard DA solutions to final concentrations of 2, 5, 10, and 20 µM. Dopamine concentrations were determined electrochemically, and the resulting responses were used to evaluate the performance of the developed electrode. All experiments were carried out in triplicate (*n* = 3) to assess reproducibility. Human urine samples were obtained from volunteer students at the University of Salerno (Italy) in accordance with institutional regulations and after obtaining informed consent.

## 3. Results and Discussion

### 3.1. Characterization of AgPt NPs

**Figure 2:**
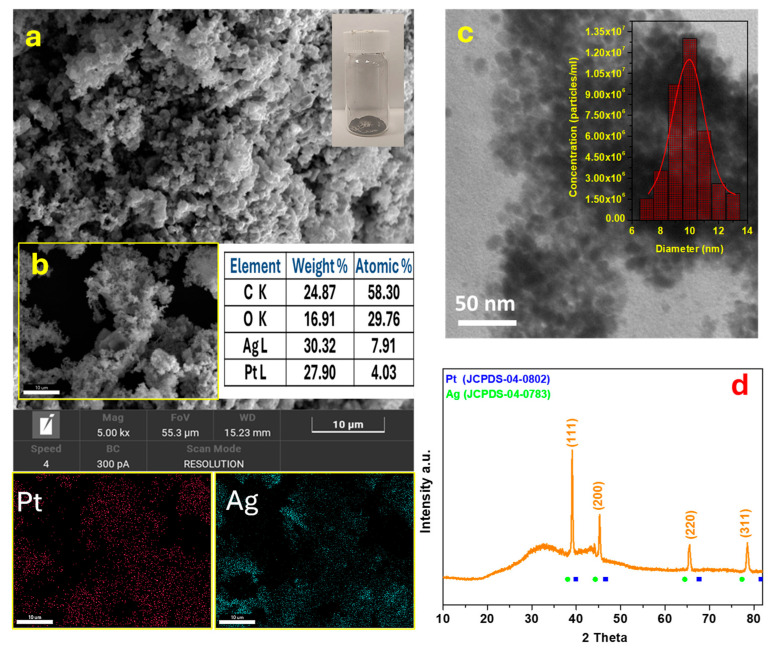
SEM images of AgPt NPs (**a**) with elemental mapping of different elements (**b**). TEM image of AgPt NPs (**c**); and XRD patterns of AgPt NPs (**d**).

The surface morphology of the bimetallic AgPt nanoparticles (NPs) was investigated using scanning electron microscopy, as shown in Figure 2a and Figure S1. The SEM images reveal the presence of nanoparticle aggregates characterized by a porous surface and a relatively uniform size distribution, with an average diameter below 500 nm. Elemental analysis, performed by energy-dispersive X-ray spectroscopy (EDX) (Figure 2b), confirmed the presence and homogeneous distribution of platinum (Pt) and silver (Ag) within the nanoparticles. The coexistence of both metals indicates successful integration into the bimetallic alloy structure. A TEM image of the AgPt nanoparticles (NPs) is presented in Figure 2c. The nanoparticles exhibit a quasi-spherical morphology and are uniformly dispersed, indicating a monodisperse size distribution. The inset in Figure 2c displays the corresponding particle size distribution histogram, derived from TEM measurements. Based on statistical analysis of multiple particles, the average particle diameter was determined to be 10.9 ± 1.21 nm. The XRD pattern shown in Figure 2d reveals several diffraction peaks located at 38.5°, 44.7°, 64.9°, and 77.1°, corresponding to the (111), (200), (220), and (311) crystal planes, respectively, of a face-centered cubic (fcc) structure. The diffraction peaks of the AgPt alloy are positioned between the standard diffraction peaks of pure Ag and Pt, suggesting the successful formation of an AgPt alloy during the synthesis process [30,31]. Furthermore, the FT-IR spectrum of the citric acid–capped AgPt alloy nanoparticles, synthesized via a solvothermal route, is shown in Figure S2. In agreement with previous studies, the presence of citrate molecules as capping agents is confirmed by the characteristic absorption peaks at 1393 cm^−1^ and 1632 cm^−1^ [32], corresponding to the symmetric and asymmetric stretching vibrations of the carboxylate (–COO^−^) groups in citrate. Additionally, the broad absorption band around 3321 cm^−1^ is attributed to the O–H stretching vibrations of hydroxyl groups present on the nanoparticle surface [33].

### 3.2. Characterization of AgPt@A-3DPE

**Figure 3:**
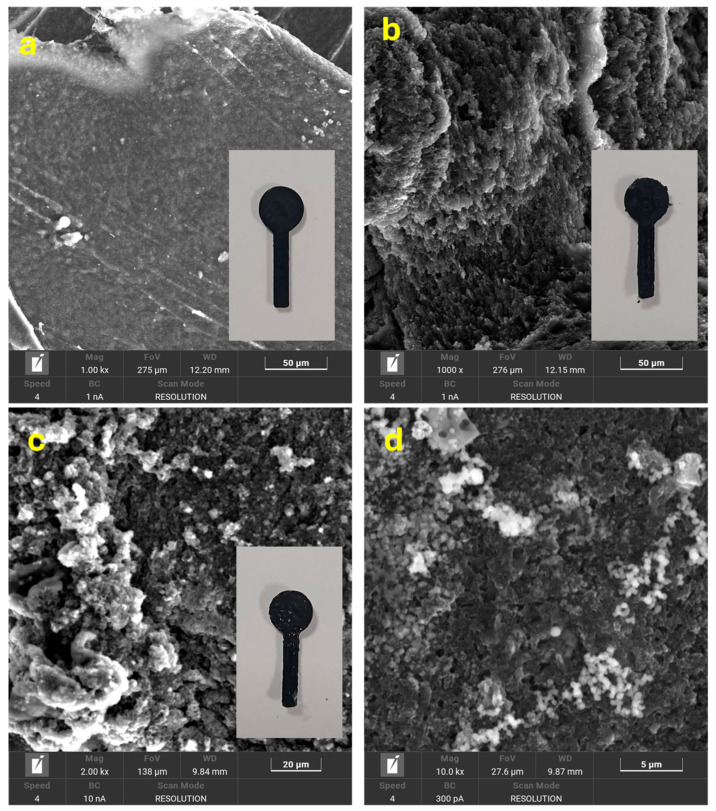
SEM images of the surface of the prepared electrodes: 3DPE (**a**), A-3DPE (**b**), and AgPt@A-3DPE (**c**,**d**).

**Figure 4:**
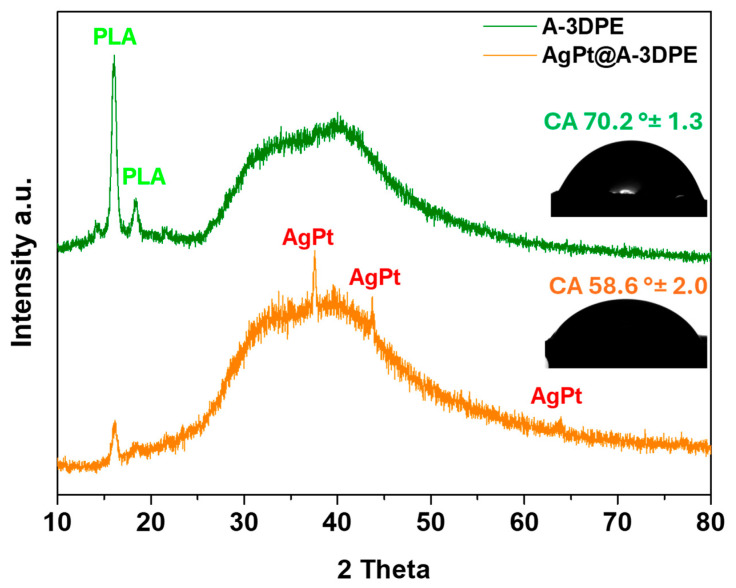
XRD patterns of the A-3DPE and Ag–Pt@A-3DPE electrodes. The inset shows the contact angle measurements of the 3D-printed electrodes, highlighting the differences in surface wettability.

The fabricated electrode materials were characterized using SEM, XRD analysis, and CA measurements. Figure 3 illustrates the surface morphologies of the 3DPE electrodes before treatment, after activation, and following coating with AgPt ink. Figure 3a and Figure S3a display the surface of the as-fabricated electrode at low and high magnifications, where no distinct carbon structures were observed on the outer layer due to the presence of non-conductive PLA. As shown in Figure 3b, after treatment with the DMF/acetone/water mixture and electrochemical activation, the surface of the 3DPE was partially etched, exposing a significant number of conductive carbon flakes, thereby yielding the activated electrode (A-3DPE). Figure 3c,d present SEM images of the electrode after modification with AgPt ink. These images revealed a rougher and more heterogeneous surface, with clearly visible agglomerates of AgPt nanoparticles distributed across the surface, suggesting that the AgPt particles successfully adhered to the A-3DPE surface. The XRD patterns of the 3D-printed electrodes after activation and subsequent AgPt ink deposition are presented in Figure 4. Distinct diffraction peaks were observed at 16.05° and 18.55°, corresponding to the characteristic crystalline planes of pure PLA [34,35]. Additionally, a broad and weak diffraction hump was detected, indicating the presence of graphitic or graphene-like structures [36]. In contrast, the XRD profile of the AgPt@A-3DPE electrode displayed additional peaks attributed to the AgPt alloy, confirming the successful deposition of the metallic nanoparticles on the activated electrode surface.

Furthermore, the wettability of the A-3DPE electrodes was evaluated at different fabrication stages, after activation and following AgPt ink modification, using contact angle measurements. As illustrated in Figure 4, the removal of the non-conductive PLA layer exposed the inherently hydrophilic carbon surface, leading to a reduced contact angle of 70.22° ± 1.3°, compared to the higher value of 82.1° ± 2.1° observed for the unmodified 3DPE (Figure S3). This reduction indicates an improvement in surface wettability, attributed to the exposure of hydrophilic carbon fibers.

Subsequent modification with AgPt ink, containing hydrophilic functional groups from citric acid-capped AgPt nanoparticles, further decreased the contact angle to 58.6° ± 2.0°, demonstrating a marked enhancement in surface hydrophilicity. This progressive reduction in contact angle correlates with the increasing hydrophilic nature of the electrode surface and further confirms the successful modification and fabrication of the AgPt@A-3DPE sensor.

### 3.3. Electrochemical Properties of AgPt@A-3DPE

**Figure 5:**
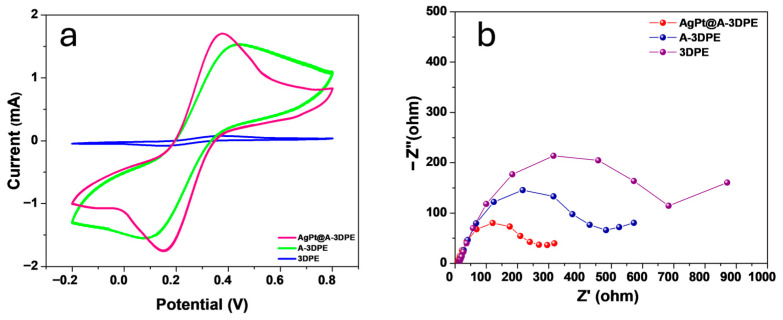
CV behavior of the 3DPE, A-3DPE, and Ag–Pt@A-3DPE electrodes recorded in 10.0 mM [Fe(CN)_6_]^3−^/^4−^ solution containing 0.1 M KCl at a scan rate of 50 mV·s^−1^ (**a**); EIS behavior of the same electrodes measured in the frequency range from 50 kHz to 0.01 Hz with an AC amplitude of ±5 mV (**b**).

**Figure 6:**
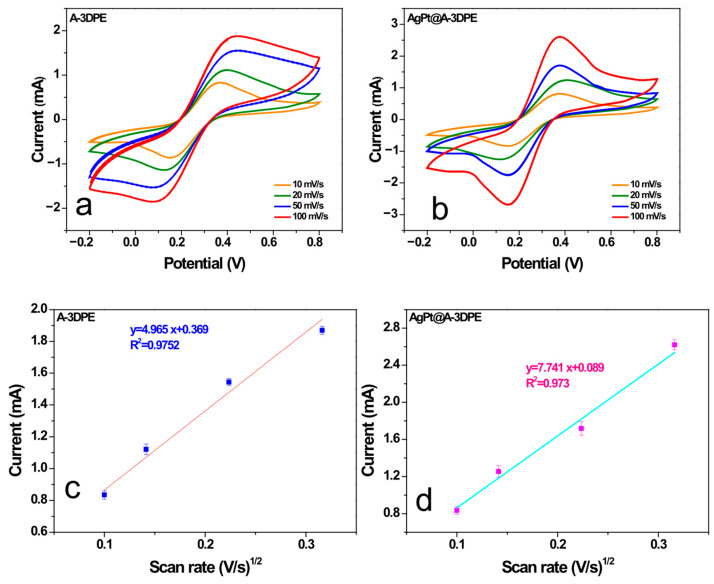
CV curves of A-3DPE (**a**) and AgPt@A-3DPE (**b**), and the corresponding calibration plots of (**c**) A-3DPE and AgPt@A-3DPE (**d**), recorded in 10 mM [Fe(CN)_6_]^4−^/^3−^ solution containing 0.1 M KCl at different scan rates (10–100 mV·s^−1^).

To investigate the electrochemical behavior of the prepared 3DPE electrodes, CV and EIS were performed. As shown in Figure 5a, the CV profile of the bare 3DPE was featureless, with no observable redox peaks for the [Fe(CN)_6_]^4−^/[Fe(CN)_6_]^3−^ couple [37,38,39,40,41], indicating poor electrical conductivity mainly due to the insulating PLA matrix. Following the activation process, the resulting A-3DPE exhibited a significant increase in redox peak currents, attributed to the removal of PLA and the exposure of conductive carbon fibers. Further modification with AgPt bimetallic nanoparticles led to an additional enhancement in electrochemical performance. Further modification with AgPt bimetallic nanoparticles led to an additional enhancement in electrochemical performance. This improvement was due to the superior properties of AgPt, including high electrical conductivity, large specific surface area, and excellent hydrophilicity, which facilitated more efficient electron transfer at the electrode surface. The electroactive surface area (A) was quantitatively determined using the Randles–Ševčík equation, applied to CV data recorded in a 0.1 M KCl solution containing the [Fe(CN)_6_]^4−^/[Fe(CN)_6_]^3−^ redox probe (Figure 6).

The equation is expressed as

Ip = 2.69 × 10^5^ × n^3/2^ × A × D^1/2^ × C × v^1/2^ (1)

where

A is the active surface area (cm^2^),
n is the number of electrons transferred (1),
D is the diffusion coefficient of potassium ferrocyanide (7.6 × 10^−6^ cm^2^/s),
C is the concentration of the redox species (0.01 M),
v is the scan rate (V/s).

As illustrated in Figure 6c,d, both A-3DPE and AgPt@A-3DPE exhibited a linear correlation between the square root of the scan rate and the anodic peak current, consistent with a diffusion-controlled and reversible electrochemical process. The calculated electroactive surface areas were 0.67 cm^2^ for A-3DPE and 1.04 cm^2^ for AgPt@A-3DPE, confirming the successful surface functionalization with AgPt nanoparticles and the enhancement of the electroactive area.

EIS analysis was conducted to further evaluate the charge-transfer properties of the electrodes, and the charge-transfer resistance (Rct) was obtained by fitting the Nyquist plots. The bare 3DPE exhibited a high Rct value of 689.0 Ω (relative standard deviation RSD = 2.6%), which decreased to 478.0 Ω (RSD = 2.8%) after activation due to the exposure of conductive carbon fibers. Upon AgPt modification, the Rct was further reduced to 292.0 Ω (RSD = 1.81%), demonstrating the superior conductivity imparted by the AgPt nanostructures.

These EIS results are in excellent agreement with the CV data, collectively confirming the progressive improvement in electrochemical performance through activation and surface modification of the electrode.

### 3.4. Effect of pH

DPV, from the standpoint of quantitative analysis, provides superior performance compared with CV, particularly for the detection of small electroactive molecules such as dopamine (DA). For this reason, it was selected and optimized in the subsequent experiments, as it allows higher sensitivity and improved peak resolution. In addition, Differential Pulse Voltammetry (DPV) plays a crucial role in minimizing interference from coexisting electroactive species. Unlike cyclic voltammetry, DPV applies a series of potential pulses superimposed on a linear potential ramp, allowing the current to be measured just before each pulse. This approach significantly reduces the contribution of capacitive (non-faradaic) currents, thereby enhancing the faradaic response associated with the oxidation of the target analyte.

As a result, DPV provides a higher signal-to-noise ratio and improved peak resolution, enabling the discrimination of electrochemical signals even when species such as dopamine, uric acid, and ascorbic acid exhibit similar oxidation potentials. This improved resolution is particularly important in complex biological matrices, where overlapping voltammetric responses are common [42].

**Figure 7:**
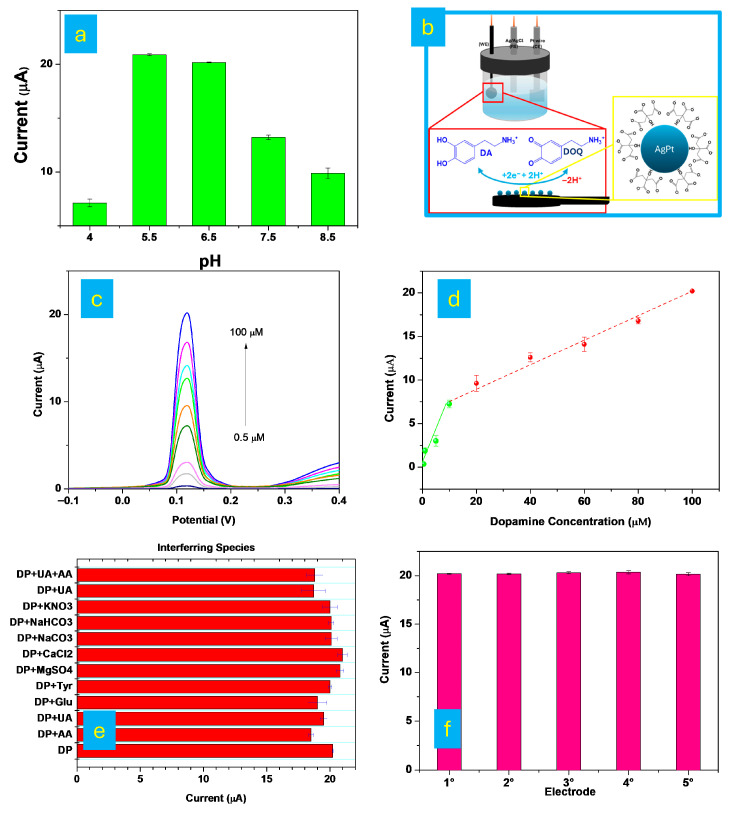
Effect of pH on the electrochemical performance of the DA sensor (**a**); proposed electrochemical sensing mechanism for DA (**b**); DPV responses of the AgPt@A-3DPE electrode toward DA concentrations ranging from 0.5 to 100.0 µM (**c**); variation in anodic peak current as a function of DA concentration within the linear ranges of 0.5–10.0 µM and 10.0–100.0 µM; (**e**) DPV responses of the AgPt@A-3DPE electrode in the presence of common interfering species along with 100.0 µM DA (**d**); and reproducibility of the AgPt@A-3DPE sensors for the detection of 100.0 µM DA (**f**).

Unlike amperometric techniques, which record a current–time response and enable the evaluation of parameters such as response time, DPV generates a current–potential response during a potential scan and is therefore particularly suitable for quantitative analysis based on the relationship between peak current and analyte concentration [42,43]. The pH of the electrolyte plays a crucial role in modulating the electrochemical response of DA at the AgPt@A-3DPE electrode. DPV measurements were performed in 0.1 M PBS at pH values ranging from 4.0 to 8.5, in the presence of 100 µM DA. As shown in Figure 7a, the peak current increased markedly as the pH rose from 5.5 to 6.5, reaching a maximum at pH 6.5. Beyond this point, under both more acidic (pH < 5.5) and more basic (pH > 6.5) conditions, a significant decrease in peak current was observed. This behavior can be explained by the pH-dependent protonation states of both DA and the electrode surface. At acidic and near-neutral pH, DA predominantly exists in its protonated, positively charged form [44]. In this state, it is electrostatically attracted to and accumulates on the negatively charged AgPt surface of the electrode, resulting in an enhanced redox response. The electrostatic interaction between protonated DA and the AgPt alloy nanoparticles facilitates efficient electron transfer, thereby amplifying the electrochemical signal (Figure 7b). However, at pH values below 5.5, carboxylic groups on the electrode surface become protonated, reducing the negative surface charge and weakening the electrostatic attraction. This diminishes DA accumulation at the electrode interface and lowers the redox response. Conversely, under basic conditions, DA becomes deprotonated and negatively charged, resulting in electrostatic repulsion from the anionic electrode surface. This repulsion hinders adsorption and oxidation, leading to a decrease in the anodic peak current. Therefore, pH 6.5 was identified as the optimal condition for DA detection, offering the highest electrochemical sensitivity due to favorable electrostatic interactions and maximized redox efficiency. In addition to the pH-dependent electrostatic interactions discussed above, the high selectivity of the AgPt@A-3DPE sensor toward dopamine can be attributed to a synergistic combination of surface chemistry and electrochemical properties. Dopamine oxidation at the electrode surface follows a well-established two-electron, two-proton transfer process, leading to the formation of dopamine-o-quinone. However, in complex biological matrices, other electroactive species such as uric acid (UA) and ascorbic acid (AA) may undergo oxidation at similar potentials, potentially causing signal overlap [45,46].

To address this challenge, the selectivity of the proposed sensor is governed by multiple concurrent mechanisms. First, at physiological pH (6.5), dopamine predominantly exists in its protonated (positively charged) form, while both UA and AA are negatively charged. The presence of carboxylate groups from the citrate-capped AgPt nanoparticles, together with the sulfonate groups of the Nafion coating, imparts a net negative charge to the electrode surface. This results in strong electrostatic attraction toward dopamine and simultaneous repulsion of negatively charged interferents.

Second, the Nafion layer acts as a cation-selective membrane, preferentially allowing the diffusion of positively charged species while effectively blocking anionic interferents such as UA and AA [47]. This selective permeability further enhances the discrimination capability of the sensor.

Finally, the AgPt bimetallic nanoparticles provide high electrocatalytic activity and facilitate faster electron transfer kinetics for dopamine oxidation, which contributes to improved signal intensity and peak definition. Similar strategies based on nanostructured materials have been demonstrated to enhance the selective detection of dopamine in the presence of interfering species [9,48]. The combined effect of electrostatic attraction, selective membrane behavior, and catalytic enhancement enables reliable and selective detection of dopamine even in the presence of common interfering species.

### 3.5. Detection of DA Using AgPt@A-3DPE Sensor

The DPV technique was applied to evaluate the electrochemical response of the sensor in the presence of DA. DPV measurements were performed over a potential range from –0.1 to 0.4 V. As shown in Figure 7c, the anodic peak current increased linearly with increasing DA concentration, within the ranges of 0.5–10 µM and 10–100 µM.

The corresponding calibration plots (Figure 7d) yielded the following equations:

I (µA) = 0.714 [DA] (µM) + 0.047 (R^2^ = 0.938) (2)

and

I (µA) = 0.147 [DA] (µM) + 6.13 (R^2^ = 0.994) (3)

The LOD was calculated using the formula LOD = 3SD/S, where SD is the standard deviation of the blank current responses, and S is the slope of the calibration curve.

This yielded a LOD of 0.037 µM and a limit of quantification (LOQ) of 0.12 µM. The AgPt@A-3DPE sensor demonstrated excellent analytical performance for dopamine detection, featuring a wide linear detection range (0.5–100 µM) and a low detection limit (0.037 µM). The enhanced electrochemical performance observed in the DPV measurements is also closely related to the improved wettability of the electrode surface after activation and AgPt modification. As demonstrated by the contact angle measurements (Figure 4), the progressive decrease in contact angle from the bare 3DPE to the AgPt@A-3DPE electrode indicates a significant increase in surface hydrophilicity. A more hydrophilic surface facilitates the diffusion of dopamine molecules from the electrolyte to the electrode interface and promotes their adsorption onto the active catalytic sites of the AgPt nanoparticles [49,50]. This improved interfacial interaction enhances the efficiency of electron transfer during the oxidation of DA, resulting in a stronger and more stable electrochemical signal in DPV. This interpretation is further supported by the observed decrease in charge-transfer resistance and increase in electroactive surface area after AgPt modification, indicating that improved wettability contributes to enhanced electron transfer kinetics. Therefore, the improved wettability of the AgPt-modified electrode contributes to the high sensitivity of the sensor, supporting the enhanced current responses observed during dopamine detection.

These results demonstrate that the developed AgPt@A-3DPE sensor is highly sensitive, reliable, and competitive with state-of-the-art electrochemical dopamine sensors reported in the literature (Table S1). In particular, the proposed sensor exhibits a wide linear response range from 0.1 to 100 μM, which is comparable to or broader than that of most previously reported systems, including graphene-based electrodes, gold nanoparticle-modified electrodes, and bimetallic nanocomposites. This broad dynamic range effectively covers both physiological and pathological dopamine concentrations, highlighting the suitability of the sensor for practical biological monitoring applications. Although the achieved detection limit (0.037 μM) is not the lowest reported, it remains highly competitive with many advanced electrochemical platforms and represents an optimal balance between sensitivity, dynamic range, and analytical robustness. Notably, these performances are obtained through a simple, scalable, and cost-effective fabrication strategy based on 3D printing combined with surface modification using AgPt nanoparticles, without the need for complex synthetic procedures or poorly reproducible materials.

### 3.6. Interference and Reproducibility

The selectivity of the AgPt@A-3DPE sensor for DA detection was evaluated using DPV in 0.1 M phosphate-buffered saline (PBS, pH 6.5) containing high concentrations of common interfering species typically present in biological fluids. The peak current of DA (100 µM) was not significantly affected by the presence of ascorbic acid (AA) or uric acid (UA), nor when both interferents were simultaneously present with DA. This behavior can be attributed to the modification of the electrode surface with citric acid, which introduces carboxylic functional groups and confers a negative charge to the electrode surface. As a result, electrostatic repulsion occurs between the negatively charged AA and/or UA species and the electrode surface, suppressing the oxidation of AA and/or UA and enhancing the selectivity toward dopamine, as also reported in the literature [50,51,52]. In addition to the Nafion layer, this surface charge effect, together with the electrocatalytic activity of AgPt nanoparticles, plays a key role in minimizing interference and improving the selective detection of dopamine.

As shown in Figure 7e, the sensor maintained a stable and selective response to DA even in the presence of additional potential interferents, including inorganic salts such as KNO_3_, NaHCO_3_, Na_2_CO_3_, CaCl_2_, and MgSO_4_, as well as organic compounds such as tyrosine (Tyr) and glucose (Glu). No significant interference was observed under any condition, confirming the high specificity of the AgPt@A-3DPE sensor for DA detection. The reproducibility of the sensor was further evaluated (Figure 7f) by independently fabricating five AgPt@A-3DPE electrodes under identical experimental conditions and testing their responses in a 100 µM DA solution. The RSD of the recorded signals was negligible, demonstrating high reproducibility and reliability of the fabrication method. Moreover, the stability of the AgPt@A-3DPE electrode was assessed using DPV measurements. As shown in Figure S4, the peak currents and integrated areas of the DPV curves for DA remained nearly unchanged after 1, 5, and 10 successive scans, indicating excellent operational stability and durability. Moreover, the long-term stability of the AgPt@A-3DPE sensor was systematically evaluated by monitoring the electrochemical response toward 100 μM dopamine in 0.1 M PBS using the same electrode over a period of one week. As illustrated in Figure S5, the recorded current responses remained highly consistent throughout the testing period, with no noticeable decrease in signal intensity. The relative standard deviation (RSD) of the measurements was calculated to be 3.88%, indicating excellent signal reproducibility. These results confirm the good stability and reliability of the fabricated AgPt@A-3DPE electrode for dopamine detection.

The AgPt@A-3DPE sensor exhibits high repeatability, reproducibility, and selectivity for dopamine detection, confirming its robust electrochemical performance for potential biosensing applications.

### 3.7. Oxidase-like Activity of AgPt NPs

The intrinsic redox activity of the AgPt NPs was qualitatively confirmed by their ability to mediate electron-transfer processes, as evidenced by the oxidation of TMB to ox-TMB. As shown in Figure S6, this reaction occurred in the absence of hydrogen peroxide and was accompanied by the appearance of a characteristic absorption band at 652 nm and a visible color change in the solution, using TMB as a chromogenic substrate. This behavior confirms evidence of the catalytic properties of AgPt NPs, supporting their role in enhancing the electrochemical performance of the AgPt@A-3DPE sensor.

### 3.8. Real Samples

Urine samples collected from different individuals were used to evaluate the applicability of the AgPt@A-3DPE electrode for the selective electrochemical detection of dopamine (DA). The urine samples were spiked with known concentrations of DA (2, 5, 10, and 20 µM) and subjected to recovery and recognition efficiency tests. The dilution of urine samples with PBS was performed to reduce matrix effects while maintaining ionic conditions like physiological environments, thereby minimizing variability due to ionic strength. In addition, potential interference from common electroactive species typically present in biological fluids, such as uric acid, ascorbic acid, glucose, and inorganic ions, was systematically evaluated in the interference study section, demonstrating negligible influence on the detection of dopamine. Well-defined oxidation peaks corresponding to DA were observed, confirming that the proposed electrode can be effectively applied for DA detection even in complex biological matrices. The recovery values obtained for DA ranged from 99.0% to 100.6% (Table 1), demonstrating excellent accuracy of the method. In addition, the relative standard deviation values were below 2.6% (*n* = 3), indicating good reproducibility. The low limit of detection (LOD = 0.037 µM) and limit of quantification (LOQ = 0.12 µM) further confirm the high sensitivity of the AgPt@A-3DPE electrode. These results demonstrate that the AgPt@A-3DPE electrode maintains reliable analytical performance even in complex biological matrices such as human urine.

## 4. Conclusions

In this study, an innovative electrochemical sensor was developed based on a 3D-printed conductive electrode modified with bimetallic AgPt nanoparticles (AgPt@A-3DPE) for the selective detection of DA. Owing to the activation treatment and nanoparticle deposition, the electroactive surface area increased from 0.67 cm^2^ (A-3DPE) to 1.04 cm^2^ (AgPt@A-3DPE). At the same time, the charge-transfer resistance (Rct) decreased from 689.0 Ω (bare 3DPE) to 292.0 Ω, highlighting a significant improvement in electrochemical performance. The sensor exhibited a linear response in two DA concentration ranges (0.5–10 µM and 10–100 µM), with correlation coefficients (R^2^) of 0.938 and 0.994, respectively. The limit of detection (LOD) and limit of quantification (LOQ) were determined to be 0.037 µM and 0.12 µM, respectively. Furthermore, the sensor maintained high selectivity in the presence of common interfering species such as ascorbic acid, uric acid, and glucose, demonstrating its suitability for complex sample analysis. Excellent reproducibility was also observed, with negligible relative standard deviation (RSD) values across five independently fabricated electrodes. The practical applicability of the AgPt@A-3DPE sensor was successfully validated through the analysis of real human urine samples. The proposed sensor achieved accurate DA quantification with recovery values close to 100% and low RSD values (<2.6%), confirming its reliability, precision, and robustness in real biological matrices. These results demonstrate that the AgPt@A-3DPE sensor is a promising, low-cost, and effective platform for dopamine determination in real-world analytical applications.

## Figures and Tables

**Table 1:** Determination of DA in a human urine sample by DPV.

Sample	Spiked (µM)	Found (µM)	Recovery (%)	R.S.D. (%)
Human urine	2	1.98	99.0	2.6
	5	4.97	99.4	2.2
	10	10.05	100.5	1.9
	20	20.12	100.6	1.6

## Data Availability

The data presented in this study are available on request from the corresponding author.

## Supplementary Materials

The following supporting information can be downloaded at https://www.mdpi.com/article/10.3390/mi17050545/s1, Table S1. Comparison of analytical performance on different electrode materials for DA detection. Figure S1. SEM images of AgPt alloy at different magnifications (a,b). Figure S2. FT-IR spectra of citric acid-capped AgPt NPs nanoparticles (a) and pure citric acid (CA) (b). Figure S3. Low-magnification SEM image of the 3DPE (a) and contact angle measurement (b). Figure S4. Stability of AgPt@A-3DE electrodes for the detection of DA. Figure S5. Stability of AgPt@A-3DE electrodes was examined by measuring 100 μM DA in 0.1 M PBS solution for one week. Figure S6. Valuation of the oxidase-like property of AgPt NPs. References [9,26,53,54,55,56,57,58,59] are cited in supplementary material.
