# Supplementary material for: Development of a 3D-Printed Nanocarbon Electrode Modified with Bimetallic Nanoparticles for Enhanced Electrochemical Detection of Dopamine
Source: Micromachines (Basel). 2026 Apr 29;17(5):545. doi: 10.3390/mi17050545 (PMC13209062; doi:10.3390/mi17050545)
Supplement: Supplementary file 1 [file micromachines-17-00545-s001.zip › micromachines-4199565-supplementary.pdf]

Supporting Information

Development of a 3D-printed nanocarbon electrode modified with bimetallic nanoparticles for enhanced electrochemical detection of dopamine

Claudia Cirillo[1,2*], Mariagrazia Iuliano[1,2], Nicola Funicello[1], Salvatore De Pasquale[1], Maria Sarno[1,2]

[1]*Department of Physics "E.R. Caianiello", University of Salerno, Via Giovanni Paolo II, 132-84084 Fisciano, Italy*

[2]*NANO_MATES Research Centre, University of Salerno, Via Giovanni Paolo II, 132-84084 Fisciano, Italy*

*Corresponding author: clcirillo@unisa.it

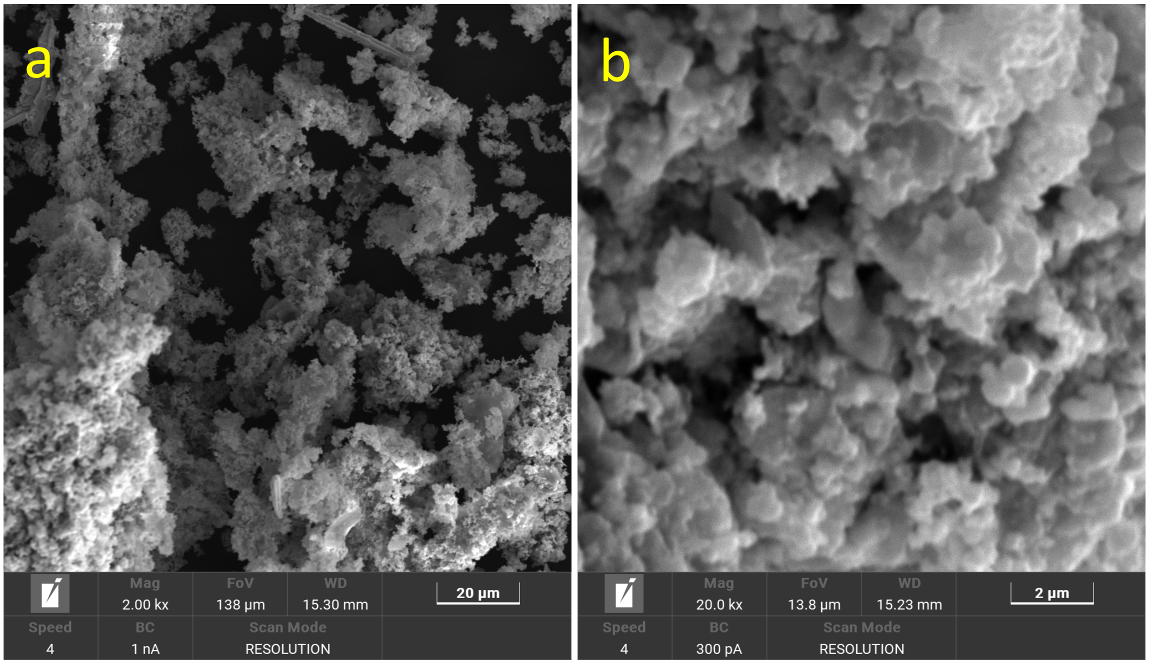

Figure S1. SEM images of AgPt alloy at different magnifications (a,b).

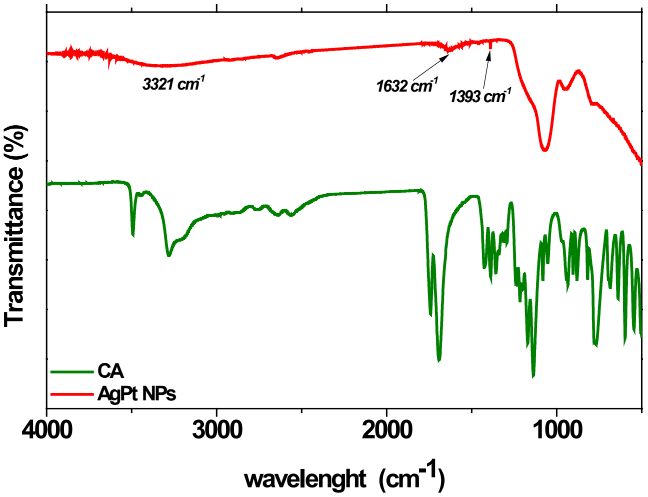

Figure S2. FT-IR spectra of citric acid-capped AgPt NPs nanoparticles (a) and pure citric acid (CA) (b).

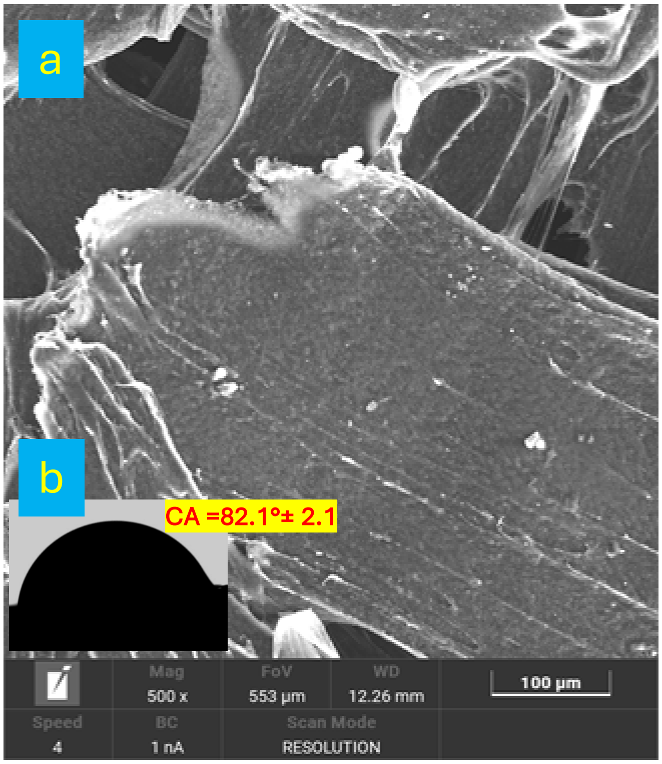

Figure S3. Low-magnification SEM image of the 3DPE (a) and contact angle measurement (b).

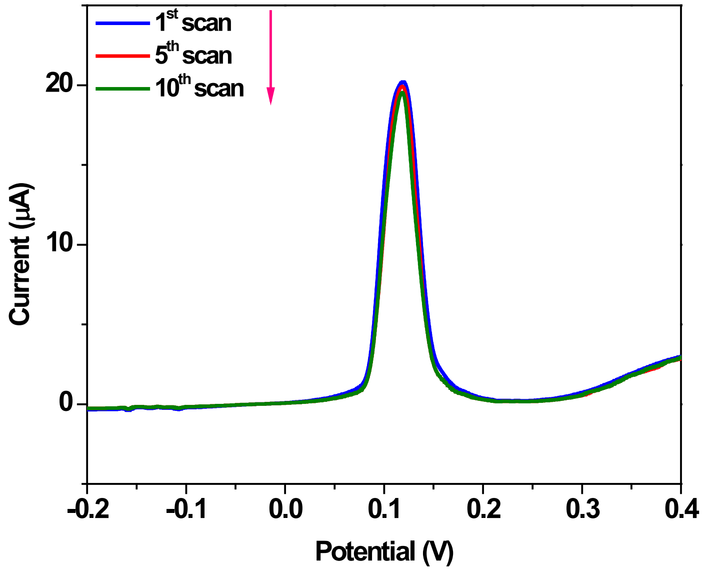

Figure S4. Stability of AgPt@A-3DE electrodes for the detection of DA.

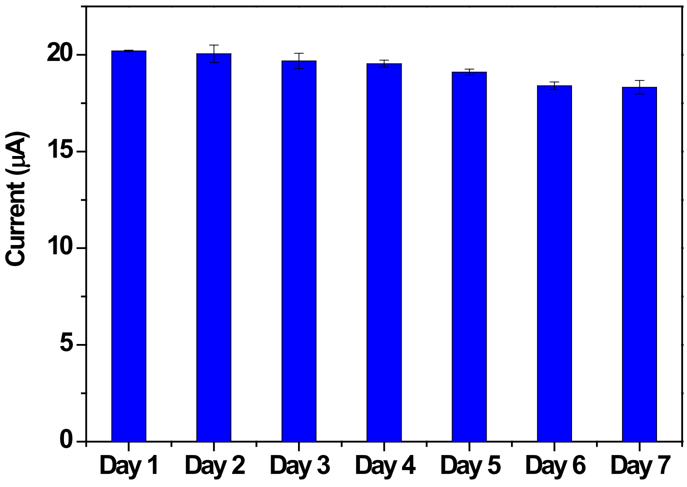

Figure S5. Stability of AgPt@A-3DE electrodes was examined by measuring 100 μM DA in 0.1 M PBS solution for one week.

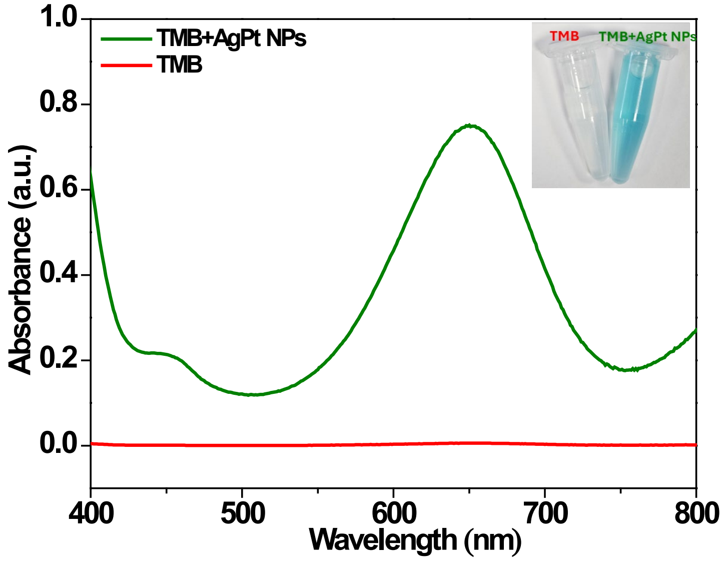

Figure S6. Valuation of the oxidase-like property of AgPt NPs.

Table S1. Comparison of analytical performance on different electrode materials for DA detection.

Electrode materials	Linear range (μM)	Detection limit (μM)	References
Au–Cu_2O/rGO	10–90	3.9	[1]
Au/PDDA/GNS	2–28	1.0	[2]
PdAu/rGO	1.25–73.75	0.75	[3]
GNP/FTO	30–100	0.22	[4]
PGE	0.2–8	0.20	[5]
MQDs@3DE	0.01–20	0.003	[6]
PANI-rGO	0.05–60	0.024	[7]
NGQDs@Au@3DE	0.00001–0.03	0.0094	[8]
Au@3DE	0.01–20	0.084	[9]
AgPt@A-3DPE	0.1–100	0.037	This work

References

[1] T. K. Aparna, R. Sivasubramanian, M. A. Dar, *One-pot synthesis of Au–Cu_2O/rGO nanocomposite based electrochemical sensor for selective and simultaneous detection of dopamine and uric acid*, **J. Alloys Compd.**, 2018, **741**, 1130–1141.

[2] H. Wang, L. G. Xiao, X. F. Chu, Y. D. Chi, X. T. Yang, *Rational design of gold nanoparticle/graphene hybrids for simultaneous electrochemical determination of ascorbic acid, dopamine and uric acid*, **Chin. J. Anal. Chem.**, 2016, **44**(12), 1617–1625.

[3] C. Zou, J. Zhong, S. Li, H. Wang, J. Wang, B. Yan, Y. Du, *Fabrication of reduced graphene oxide-bimetallic PdAu nanocomposites for the electrochemical determination of ascorbic acid, dopamine, uric acid and rutin*, **J. Electroanal. Chem.**, 2017, **805**, 110–119.

[4] M. M. Rahman, N. S. Lopa, M. J. Ju, J. J. Lee, *Highly sensitive and simultaneous detection of dopamine and uric acid at graphene nanoplatelet-modified fluorine-doped tin oxide electrode in the presence of ascorbic acid*, **J. Electroanal. Chem.**, 2017, **792**, 54–60.

[5] Y. Wang, Y. Huang, B. Wang, T. Fang, J. Chen, C. Liang, *Three-dimensional porous graphene for simultaneous detection of dopamine and uric acid in the presence of ascorbic acid*, **J. Electroanal. Chem.**, 2016, **782**, 76–83.

[6] M. Wan, A. Jimu, H. Yang, J. Zhou, X. Dai, Y. Zheng, J. Ou, Y. Yang, J. Liu, L. Wang, *MXene quantum dots enhanced 3D-printed electrochemical sensor for the highly sensitive detection of dopamine*, **Microchem. J.**, 2022, **184**, 108180. https://doi.org/10.1016/j.microc.2022.108180

[7] L. Q. Xie, Y. H. Zhang, F. Gao, Q. A. Wu, P. Y. Xu, S. S. Wang, N. N. Gao, Q. X. Wang, *A highly sensitive dopamine sensor based on a polyaniline/reduced graphene oxide/Nafion nanocomposite*, **Chin. Chem. Lett.**, 2017, **28**, 41–48.

[8] L. Zhong, X. Du, Y. Jiang, J. Wen, X. Wang, S. Wang, R. Peng, M. Liao, J. Ou, Y. Yang, L. Wang, *N-doped graphene quantum dots and gold co-modified 3D printed electrode for sensitive detection of dopamine*, **Microchem. J.**, 2025, 113432. https://doi.org/10.1016/j.microc.2025.113432

[9] L. Zhong, M. Liao, J. Ou, Y. Yang, J. Wen, Y. Jiang, H. Yang, X. Dai, L. Wang, *Gold particles modified 3D printed carbon black nanonetwork electrode for improving the detection sensitivity of dopamine*, **Microchem. J.**, 2024, **201**, 110630. https://doi.org/10.1016/j.microc.2024.110630
